# Dietary Magnesium Intake Affects the Vitamin D Effects on HOMA-β and Risk of Pancreatic β-Cell Dysfunction: A Cross-Sectional Study

**DOI:** 10.3389/fnut.2022.849747

**Published:** 2022-03-29

**Authors:** Rongpeng Gong, Yuanyuan Liu, Gang Luo, Lixin Yang

**Affiliations:** ^1^Medical College of Qinghai University, Xining, China; ^2^Endocrinology Department, Qinghai Provincial People's Hospital, Xining, China; ^3^College of Eco-Environmental Engineering, Qinghai University, Xining, China

**Keywords:** Magnesium intake, vitamin D, HOMA-β index, pancreatic β-cell, cross-sectional study

## Abstract

**Background:**

Some studies have shown that, the circulating vitamin D (Vit D) concentration in the body exerts a crucial role in regulating the pancreatic β-cell function. Meanwhile, the role of magnesium is important in the synthesis of Vit D, since it is an essential element for activating Vit D. Nevertheless, there remains insufficient studies concerning whether dietary Magnesium intake influences the association between Vit D and risk of pancreatic β-cell dysfunction. Hence, this cross-sectional study aimed to assess the effect of Magnesium intake alterations on the association between serum Vit D levels and the risk of pancreatic β-cell dysfunction.

**Methods:**

This large-scale cross-sectional study involves four cycles of National Health and Nutrition Examination Survey (NHANES) (2007–2014), with totally 4,878 participants. Groups were divided depending on the median daily intake of Magnesium, namely, the low intake group (Magnesium intake <267 Magnesium/d) and the high intake group (Magnesium intake ≥ 267 Magnesium/d). By constructing multiple multivariate linear and logistics regression models, the associations between serum Vit D levels and HOMA-β, as well as between serum Vit D levels and the risk of pancreatic β-cell dysfunction were explored at different Magnesium intakes.

**Results:**

In this cross-sectional study, the serum Vit D level is independently correlated with the HOMA-β index [β: 0.65 (0.40–0.90)] and the risk of pancreatic β-cell dysfunction [OR: 0.95 (0.92–0.98)]. Moreover, such correlations are affected by different dietary Magnesium intakes (*P* for interaction < 0.001).

**Conclusion:**

According to the results of this study, the dietary Magnesium intake influences the associations of serum Vit D levels with HOMA-β index and pancreatic β-cell dysfunction. Besides, the finding requires validation through more RCT or cohort studies.

## Background

Pancreatic β-cells refer to a kind of insulin-secreting cells of the pancreas. Both impaired pancreatic β-cell functions and relatively insufficient insulin secretion can increase the blood sugars (1). Based on surveys, the impairment of pancreatic β-cells has become a ubiquitous phenomenon worldwide, and most patients with pancreatic β-cell impairment are highly likely to progress to diabetes without intervention or control (2–4). In addition, it has been reported that one of the main pathogeneses of type 2 diabetes is impairment of pancreatic β-cells. The International Diabetes Federation (IDF) claimed that 629 million people aged 20–79 will be estimated to have diabetes by 2045, occupying ~10% of the total global population (5, 6). This implies that, there may be more patients with impaired pancreatic β-cells hiding under this population. This is a huge challenge to the world health and medical resources. If the pancreatic β-cell impairment can be detected in advance, it will have a profound significance for the occurrence and prevention of diabetes. At the same time, for medical researchers, preventing the pancreatic β-cell damage and reducing the diabetes incidence are an incumbent responsibility. This highly necessitates the identification of the effects of nutrients on β-cell functionality and the prevention of pancreatic β-cell impairment.

Vitamin D (Vit D), as a kind of lipid-soluble vitamins, has vitamins D3 and D2 as major components (7). According to some research findings, Vit D can increase the blood calcium and phosphorus while regulating the bone metabolism in coordination with parathyroid hormones, exhibiting preferable performance in enhancing immunity, as well as in preventing cancer, cardiovascular and metabolic diseases (8–12). However, its association with pancreatic β-cell functionality remains controversial. Most studies have claimed that Vit D can improve the functionality of pancreatic β-cells and insulin sensitivity, while lowering the risk of type 2 diabetes(T2DM) (12–17). Contrastively, other studies have shown that Vit D does not affect β-cell functionality, nor is it a risk factor of cardiovascular diseases, which is even unrelated to diabetes (18–21). Based on an RCT study conducted by Nielsen et al. among Greenland Inuit in 2015, Vit D may be uncorrelated with the risk of T2DM, albeit the presence of a negative correlation between Vit D levels and pancreatic β-cell functions (22). The reason for these disparities in the foregoing results may be that the pancreatic β-cell functionality is affected concurrently by factors such as race, region, age and gender, one of which is the dietary Magnesium intake.

Magnesium is the fourth most abundant mineral in the human body after calcium, potassium and sodium. Magnesium can activate ~600 enzymes, which are essential for maintaining cellular stability, synthesizing RNAs and DNAs, repairing cells, and sustaining the cellular antioxidant status (23, 24). There exists a close association between Magnesium and synthesis of Vit D, and previous research has demonstrated that Magnesium is an essential element for activating Vit D (24). Neither vitamin D2 or D3 has bioactivity, which needs to be hydroxylated twice in the liver and kidney, in order to produce active 1,25(OH)2D. The enzymatic activities of hepatic 25-hydroxylase and renal 1α-hydroxylase are both Magnesium-dependent processes (25, 26). Some other studies have revealed that Vit D is transported in the human body in conjunction with carrier proteins, where the transport carrier is Vit D-binding protein and that the activity of such Vit D-binding protein is also a Magnesium-dependent process (27, 28). According to a review by Kostov et al. Magnesium plays a crucial role in regulating the electrical activity of pancreatic β-cells and insulin concentration. This may be attributed to the associations of intracellular Magnesium concentration with the phosphorylation of target cell insulin receptors and downstream signal kinases (29). By summarizing previous studies, Toi et al. (30) concluded that the substantial Magnesium intake is negatively correlated with the risk of pancreatic β-cell impairment.

Can Magnesium, as an activator of Vit D and a regulator of pancreatic β-cell functionality, affect the association between serums Vit D levels and pancreatic β-cells? There have been few similar reports in the existing literature (8, 31, 32). Hence, clinical research concerning the effect of Magnesium intake on the association of serums Vit D levels with the risk of pancreatic β-cell impairment is necessary. In this study, we hypothesize that Magnesium intake can affect such association. The objective is to explore the effect of Magnesium intake on the association between Vit D and risk of pancreatic β-cell impairment by adopting a nationally representative public database of the USA.

## Methodology

### Data Source

This large cross-sectional study utilizes four cycles (2007–2014) of data from the National Health & Nutrition Examination Survey (NHANES) database (https://www.cdc.gov/nchs/nhanes/). As a research project related to the diet and health of the US citizens, a multistage stratified probability design is adopted during data collection. Therefore, the samples are representative of the entire US citizens who are not in shelter institutions. These data include demographic, dietary, somatometric, laboratory and questionnaire data. All the NHANES-based study protocols have been approved by the Research Ethics Review Board of National Center for Health Statistics (NCHS). Ethical approval and more detailed information can be found on the Review Board's website (https://www.cdc.gov/nchs/nhanes/irba98.htm) (33).

### Study Design and Participants

This study was designed as a cross-sectional study, where the target independent variable was the serum Vit D level at the time of participant testing, and the dependent variable was whether the participants were diagnosed with pancreatic β-cell dysfunction. Depending on the median daily intake of Magnesium, the participants were divided into the low intake group (Magnesium intake <267 Magnesium/d) (*n* = 2,436) and the high intake group (Magnesium intake ≥ 267 Magnesium+/d) (*n* = 2,442) (8).

In the current work, participants aged above 20 who completed interviews and exams at the Mobile Examination Center (MEC) between 2007 and 2014 were recruited. Those who satisfied the following criteria were excluded: (1) Participants lacking information about serum Vit D concentration, fasting plasma glucose (FPG) and insulin measurements. (2) Patients taking drugs that affect glucose or interfere with β-cell functionality. (3) Those with combined hepatobiliary and renal diseases or diseases that affect glucose metabolism. (4) Any history of osteoporosis or other bone metabolism abnormalities. (5) Patients with any recent history of surgery, trauma or serious illness, e.g., a history of stress. (6) Serious diseases, such as malignant tumors (34).

### Data Collection

All data were collected by well-trained professionals. The data used in this study included demographics (age, gender, race, education, etc.), anthropometric measurements (height, waist circumference, weight, BMI, etc.), health-related behaviors (smoking and exercise) and biochemical tests (FPG, OGTT, etc.). All information and blood samples were collected in the MEC. The basic information was collated immediately, while the serum samples were scientifically stored and subsequently sent to the NCHS laboratory of The Centers for Disease Control and Prevention (CDC) and designated agencies for analysis (35).

#### Magnesium Intake Measurement

The Magnesium intake protocol adopted in this study is based on the consensus reached at the expert evaluation program seminars that are regularly held by NHANES. During the large cross-sectional study, the dietary intake was determined by 24 h dietary recall. In this study, data on dietary Magnesium intake in the first 24 h were acquired through MEC's diet recall interviews. In accordance with the median value (267 Magnesium+/day), the daily Magnesium intake was classified as either high or low.

#### Vit D Measurement

Following the MEC serum sampling, the samples were immediately frozen and stored at−30°C. Then, the samples for Vit D measurement were uniformly transported to the CDC's Environmental Health Laboratory in Atlanta, Georgia. The Vit D level was defined as the sum of Vit D3 and D2. In addition, the ultra-high performance liquid chromatography–tandem mass spectrometry (UHPLC-MS/MS) was employed as the laboratory analysis method (36).

#### Diagnosis of Pancreatic β-Cell Dysfunction and HOMA-β

HOMA-β index is considered a good indicator for evaluating the β-cell functionality (37, 38). The computational formula for HOMA-β is: 20 × fasting insulin (FINS) level (μU/mL)/FPG level (mmol/L)-3.5(%) (%). In this study, the β-cell dysfunction was identified by whether the HOMA-β index was lower than 75% of participants. Through the calculation, this value was found to be 61.76. Accordingly, the pancreatic β-cells were regarded as dysfunctional when HOMA-β <61.76, and intact when HOMA-β ≥ 61.76.

#### Definition of Some Other Variables

##### Diabetes

By multiplying FPG by 0.056 (rounded to three decimal places), the unit was converted from Magnesium/dl to mmol/l (35). The diagnostic criteria for diabetes included: FPG 7.0 mmol/l, OGTT 11.1 mmol/l, doctor diagnoses, self-reports or diabetes medication intakes.

##### Races

Mexican Americans, other Hispanics, non-Hispanic whites, non-Hispanic blacks, and other races.

##### Educational Levels

Middle school, senior high school, university or above.

##### Smoking

Currently smoking, quit smoking, and never smoked. Participants who smoked ≥100 cigarettes in total in the past and reported that they had smoked for several days or every day during the interviews were considered current smokers. Participants who have smoked <100 cigarettes in the past but do not currently smoke were considered smoking quitters. Participants who smoked <100 cigarettes in the past were considered non-smokers.

##### Sports Activities

Walking, moderate- and high-intensity activities.

##### BMI

Calculated based on height and weight. Heights were measured by research staff with an electronic stadiometer (Seca Ltd., Medical Scales and Measurement Systems, Birmingham, UK) to the nearest ml. Weights were measured by research staff on a digital scale (Mettler-Toledo, LLC, Columbus, OH, USA). After the completion of the measurements, pounds were converted into kg. The formula for BMI is: BMI = weight (kg)/height (M2) (39). The dietary data came from diet recall interviews in the first 24 h, including total dietary energy, Vit D, calcium, magnesium, protein and fiber.

### Statistical Methods

Every year, NHANES selects 5,000 people from its sampling frames over 15 different locations in all the US counties. Thus, its data is representative of a broad American population. To prevent the deviation and inaccuracy of estimation results caused by oversampling of minority groups, we adopted a weight recommended by NHANES, indicating that all our research analyses below are based on weighted model.

All data were analyzed using R version 4.1.2. Continuous variables were represented through detailed sample descriptions, with an average confidence interval of 95%. Categorical variables were represented by counts and weighted percentages. Skew distributions were based on the medians and Q1–Q3, while normal distributions were described by the mid-values and standard deviations. Inter-group comparisons of continuous variables were made by normality-based Student *t* test or Mann-Whitney U test, while intra-group comparisons were performed by Fisher's exact probability method. Covariate selection was based on potential confounders that might be associated with the functionalities of Vit D and pancreatic β-cells. Based on previous literature, international standards and relevant clinical experiences, we chose gender, age, race, smoking, BMI, obesity, dietary intake, physical activity and educational level as covariates after comprehensive consideration. The purpose of filling in missing covariates by multiple imputation is to maximize statistical power and minimize bias. Besides, sensitivity analysis was performed to observe whether the generated complete data differed significantly from the original data. The results demonstrate that the multiple imputed data differ statistically insignificantly from the original data (*P* > 0.05). Thus, according to the Rubin criterion, all the results of our multivariate analysis are based on the multiple imputed datasets.

*P*-values < 0.05 (two-sid Three multivariate linear regression models were built to analyze the association between Vit D and HOMA-β in samples at different Magnesium intakes. Meanwhile, smooth fitting curves were constructed.) were considered statistically significant. In addition, we also developed three multivariate logistic regression models to analyze the association of Vit D with pancreatic β-cell dysfunction under different Magnesium intakes. To ensure the analytical robustness, sensitivity analysis was carried out. Vit D was converted into a categorical variable, and the *P*-value for the trend was calculated. The purpose was to observe whether there was a possibility of non-linearity between Vit D and β-cell functionality when the Vit D level was regarded as a categorical variable.

## Results

### Description of Basic Demographic Information

**Figure 1:**
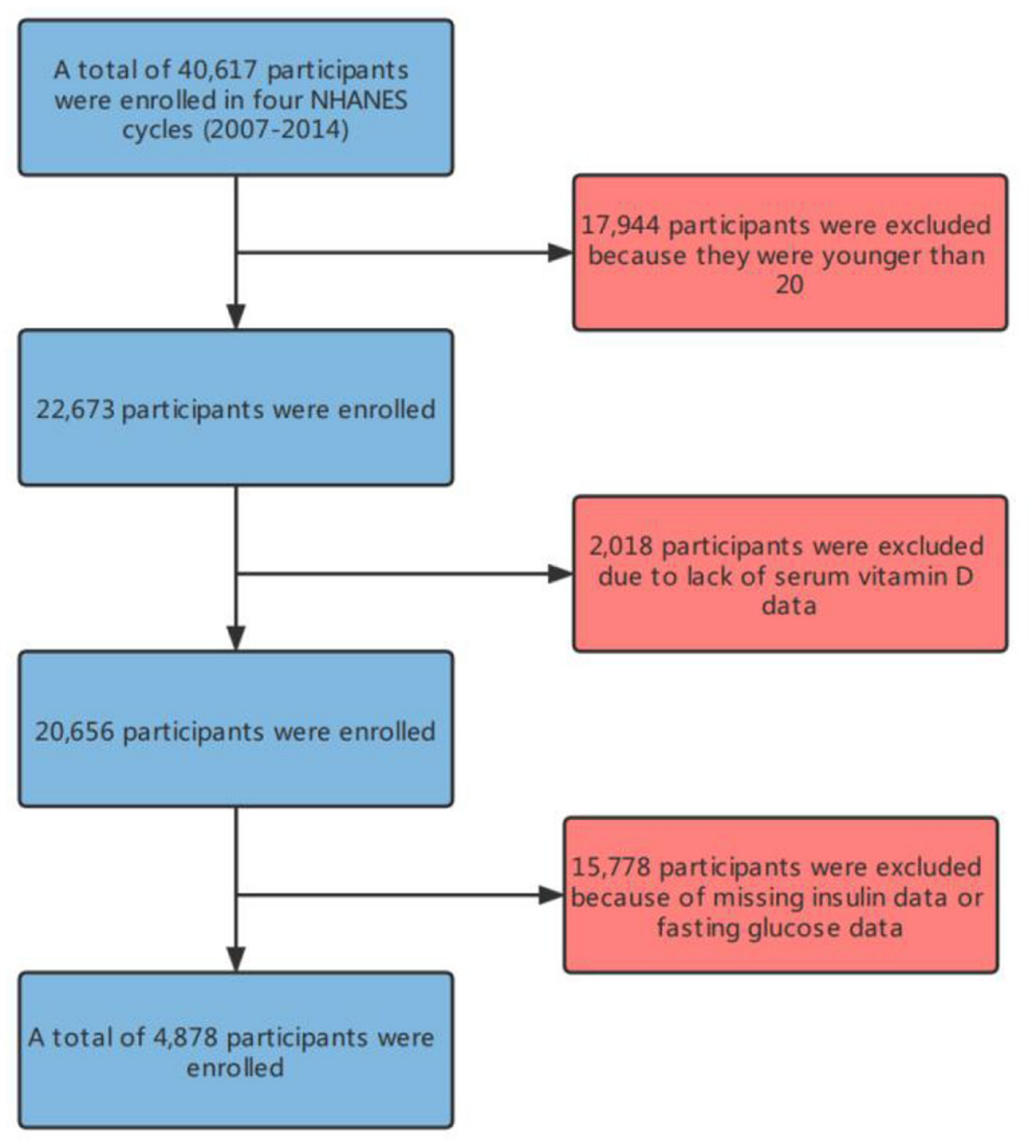
Flowchart of participant selection.

**Figure 2:**
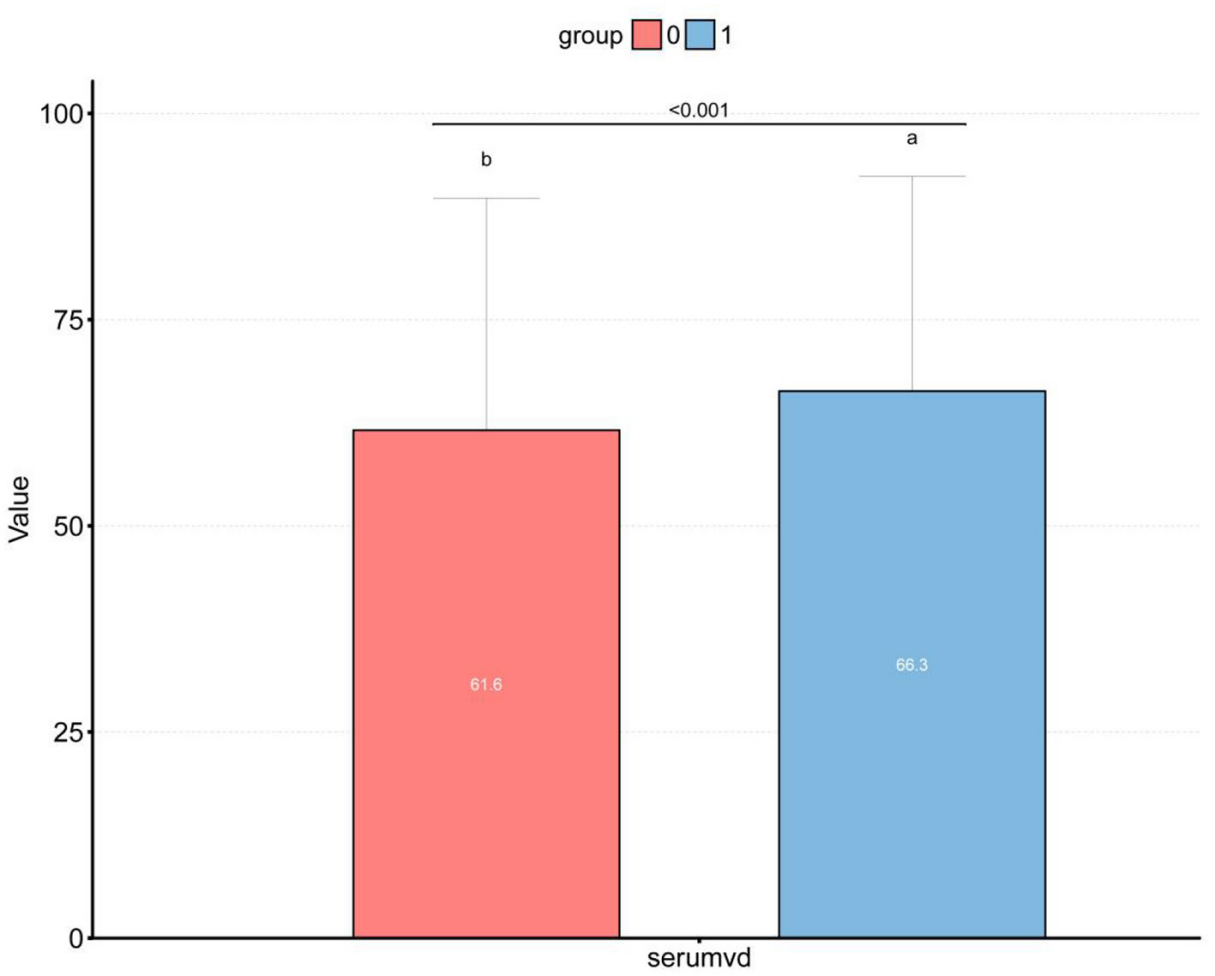
Box plot of the differences between different vitamin D levels in the high and low dietary Magnesium intake groups. **(a)** 1, high dietary Magnesium intake group; **(b)** 0, low dietary Magnesium intake group; *p* < 0.001.

In this study, there were 4,878 participants (Figure 1) in the four cycles of NHANES (2007–2014). Table 1 details the basic information of the enrolled participants. Groups were divided depending on the Magnesium intake, namely, the low intake group (<267 Magnesium+/d) and the high intake group (≥267 Magnesium+/d) (Figure 2).

**Table 1:** Basic information description of participants.

**Variables**	**Total** **(*n* = 4,878)**	**Dietary Magnesium intake (mg/d)**		***P*-value**
		**<267 mg/d** **(*n* = 2,436)**	**≥267 mg/d** **(*n* = 2,442)**	
Age, mean ± SD	49.2 ± 17.7	50.3 ± 18.4	48.0 ± 16.9	<0.001
BMI, mean ± SD	28.9 ± 6.8	29.2 ± 7.0	28.6 ± 6.6	0.004
FPG, mean ± SD	6.0 ± 1.9	6.0 ± 1.9	5.9 ± 1.9	0.406
OGTT, mean ± SD	7.7 ± 4.3	7.9 ± 4.3	7.4 ± 4.2	<0.001
HOMA-β	97.4 (61.8, 150.4)	100.6 (63.2, 153.4)	94.2 (59.8, 147.4)	0.016
Serum-Vd, median (IQR)	61.4 (44.2, 79.3)	58.6 (41.1, 77.0)	63.8 (48.0, 81.5)	<0.001
Gender, *n* (%)				<0.001
Male	2,353 (48.2)	947 (38.9)	1,406 (57.6)	
Female	2,525 (51.8)	1,489 (61.1)	1,036 (42.4)	
Race, *n* (%)				<0.001
Mexican American	716 (14.7)	294 (12.1)	422 (17.3)	
Other Hispanic	477 (9.8)	243 (10)	234 (9.6)	
Non-Hispanic white	2,307 (47.3)	1,139 (46.8)	1,168 (47.8)	
Non-Hispanic black	913 (18.7)	544 (22.3)	369 (15.1)	
Other races	465 (9.5)	216 (8.9)	249 (10.2)	
Obesity, *n* (%)				0.001
No	3,134 (64.2)	1,510 (62)	1,624 (66.5)	
Yes	1,744 (35.8)	926 (38)	818 (33.5)	
Education, *n* (%)				<0.001
Did not graduate from high school	1,228 (25.2)	688 (28.2)	540 (22.1)	
Graduated from high school	1,068 (21.9)	600 (24.6)	468 (19.2)	
College education or above	2,582 (52.9)	1,148 (47.1)	1,434 (58.7)	
Activity, *n* (%)				0.772
Vigorous work activity	899 (18.4)	464 (19)	435 (17.8)	
Moderate work activity	1,032 (21.2)	505 (20.7)	527 (21.6)	
Walk or bicycle	682 (14.0)	343 (14.1)	339 (13.9)	
Vigorous recreational activities	330 (6.8)	168 (6.9)	162 (6.6)	
Moderate recreational activities	1,935 (39.7)	956 (39.2)	979 (40.1)	
Diabetes, *n* (%)				<0.001
No	3,905 (80.1)	1,892 (77.7)	2,013 (82.4)	
Yes	973 (19.9)	544 (22.3)	429 (17.6)	
Season of examination, *n* (%)				0.327
Winter	2,304 (47.2)	1,133 (46.5)	1,171 (48)	
Summer	2,574 (52.8)	1,303 (53.5)	1,271 (52)	
Dietary factors				
Energy (kcal)	2106.1 ± 10.3	1582.5 ± 6.7	2628.4 ± 15.1	<0.001
Protein (gm)	81.9 ± 42.9	59.1 ± 24.8	104.6 ± 45.0	<0.001
Fiber (gm)	16.7 ± 10.3	10.7 ± 5.1	22.7 ± 10.7	<0.001
Calcium (mg)	920.7 ± 603.4	639.1 ± 342.2	1201.6 ± 672.7	<0.001
Beta cell function is impaired				<0.001
No	3,658 (75.0)	1,856 (76.2)	1,802 (73.8)	
Yes	1,220 (25.0)	580 (23.8)	640 (26.2)	

*BMI, Body Mass Index; FPG, Fasting plasma glucose; OGGT, Oral Glucose Tolerance Test*.

For all participants, their mean age was 49.2 ± 17.7 years. The low intake group exhibited a mean age of 50.3 ± 18.4 years, while the high intake group showed a mean age of 48.0 ± 16.9 years, presenting a significant inter-group difference (*P* < 0.001). Among obese population, the proportion of participants increased in the low intake group (35.8% → 38%), while decreased in the high intake group (35.8% → 33.5%). The BMI, FPG and OGTT levels were all higher in the low intake group than those in the high intake group. Contrastively, the Vit D level in the high intake group was significantly higher than that in the low intake group (Table 1).

### Distribution of Serum Vitamin D in Pancreatic β-Cell Dysfunction Grouped by Magnesium Intake

**Figure 3:**
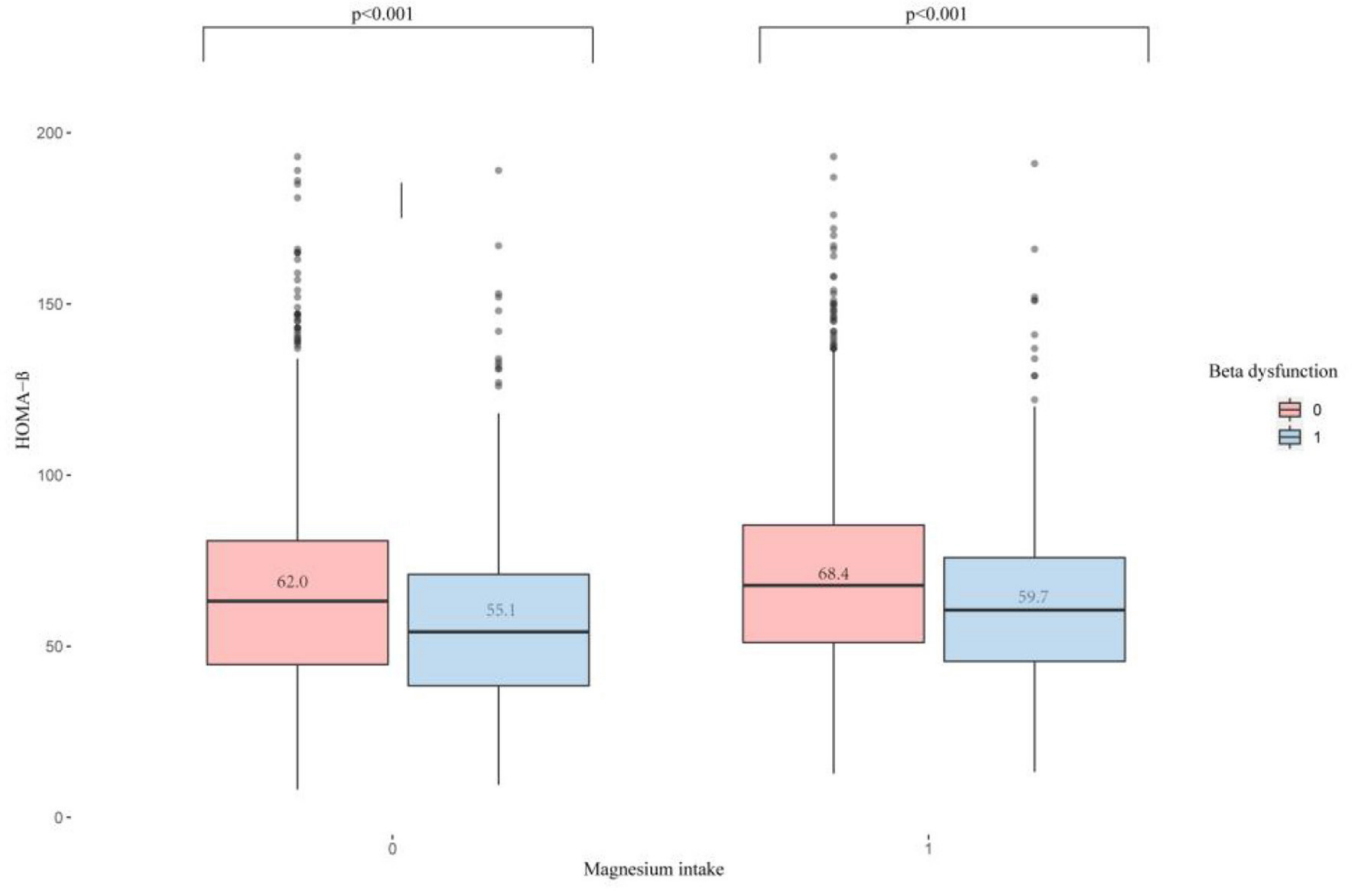
Distribution of serum Vitamin D in pancreatic β-cell dysfunction grouped by Magnesium intake. 0, IR-positive; 1, IR-negative; *p* < 0.001.

Figure 2 displays the difference in Vit D level between high and low Magnesium intake groups (61.6 vs. 66.3 nmol/L, *P* < 0.001). Meanwhile, we observed that the Vit D level differed among the β-cell dysfunction-positive and -negative groups (*P* < 0.001), as shown in Figure 3. The dysfunction-positive group exhibited significantly lower Vit D levels than the negative group (high Magnesium intake group: 59.7 vs. 68.4 nmol/L, *P* < 0001; low Magnesium intake group: 55.1 vs. 62.0 nmol/L, *P* < 0.001).

### Univariate Regression Analysis

Univariate logistic regression was adopted for analyzing which factors might be correlated with the risk of pancreatic β-cell dysfunction. As illustrated in Table 2, age, gender, race, obesity, educational level, serum Vit D level and Magnesium intake were all correlated with the risk of pancreatic β-cell dysfunction. Considering the potential influences of the above variables on main results, we controlled these factors as covariates in our main analysis.

**Table 2:** Association of covariates and impaired pancreatic β-cell function.

**Variable**	**OR (95%CI)**	***P*-value**
Age	1.02 (1.02–1.02)	<0.001
Gender, *n* (%)		
Male	1	
Female	0.69 (0.61–0.79)	<0.001
Race/ethnicity, n (%)		
Mexican American	1	
Other Hispanic	1.18 (0.89–1.58)	0.254
Non-Hispanic white	1.65 (1.34–2.04)	<0.001
Non-Hispanic black	1.46 (1.15–1.86)	0.002
Other races	1.94 (1.48–2.56)	<0.001
Obesity, *n* (%)		
No	1	
Yes	6.73 (5.9–7.67)	<0.001
Education level, *n* (%)		
Did not graduate from high school	1	
Graduated from high school	0.98 (0.81–1.18)	0.839
College education or above	0.95 (0.81–1.11)	0.48
Smoking status, *n* (%)		
Current smoker	1	
Former smoker	0.93 (0.77–1.13)	0.468
Never smoker	1.06 (0.9–1.25)	0.468
Physical activity, *n* (%)		
Vigorous work activity	1	
Moderate work activity	1.28 (1.04–1.58)	0.02
Walk or bicycle	1.46 (1.16–1.83)	0.001
Vigorous recreational activities	1.23 (0.92–1.65)	0.161
Moderate recreational activities	1.08 (0.89–1.3)	0.439
Season of examination, *n* (%)		
Winter	1	
Summer	0.91 (0.81–1.02)	0.099
Serum-Vd	0.99 (0.99–0.99)	<0.001
Ma-intake	0.99 (0.99–0.99)	<0.001

*Data presented are ORs and 95% Cls*.

### Multivariate Regression Analysis

In the present study, three linear regression models were built to analyze the independent correlation between Vit D level and HOMA-β index, and to clarify whether such correlation was influenced by different levels of Magnesium intake. Table 3 details the effect sizes β and 95% CIs. The model-based effect sizes indicate that for every additional unit of Vit D, the HOMA-β index increases correspondingly. For example, the total effect size in the unadjusted model is 1.38. Every additional unit of Vit D implies a corresponding increase in the HOMA-β index by 0.38. In the high intake group, the effect size β and 95% CI were 0.40 (1.23–1.57), while in the low intake group, the odds ratio (OR) and 95% CI were 0.32 (1.17–1.47). In terms of Model 2, only the sociodemographic data was adjusted, which yielded total effect size β and 95% CI of 0.42 (0.20–0.63). In the high intake group, the OR and 95% CI were 0.44 (0.20–0.63), while in the low intake group, the effect size β and 95% CI were 0.38 (0.20–0.56). For the fully adjusted Model 3, the total effect size β and 95% CI were 0.65 (0.40–0.90). In the high intake group, the OR and 95% CI were 0.64 (0.39–0.89), while in the low intake group, the effect size β and 95% CI were 0.67 (0.40–0.94). Based on the above results, Vit D level and HOMA-β index are independently correlated, and such correlation is affected by the level of Magnesium intake.

**Table 3:** Interactive effect of vitamin D and dietary magnesium intake on HOMA-β.

**Variable**	**Model 1**			**Model 2**			**Model 3**		
	**β (95%CI)**	***P*-value**	***P* for interaction**	**β (95%CI)**	***P*-value**	***P* for interaction**	**β (95%CI)**	***P*-value**	***P* for interaction**
VD	0.38 (0.20–0.55)	<0.001		0.42 (0.20–0.63)	<0.001		0.65 (0.40–0.90)	<0.001	
**Magnesium intake group**									
<267 mg/day VD	0.32 (0.07–0.47)	<0.001	<0.001	0.38 (0.20–0.56)	<0.001	<0.001	0.64 (0.39– 0.89)	<0.001	<0.001
≥267 mg/day VD	0.40 (0.23–0.57)	<0.001		0.44 (0.20–0.63)	<0.001		0.67 (0.40–0.94)	<0.001	

*Model 1, Non-adjusted; Model 2, adjusted age, gender, race; Model 3, Adjusted age, gender, race, obesity, education level, physical activity, smoking status, the season of examination, and dietary calcium intake*.

In addition, we also built three logistic regression models to analyze the independent correlation between Vit D level and pancreatic β-cell dysfunction, and to clarify whether such correlation was affected by the level of Magnesium intake. Table 4 details the effect size ORs and 95% CIs. The model-based effect sizes demonstrate that after adjusting the covariates based on the complete model (Model 3), the Vit D level was independently correlated with the pancreatic β-cell dysfunction and was affected by the level of Magnesium intake. The overall effect size OR and 95% CI were 0.95 (0.92–0.98). In the high intake group, the OR and 95% CI were−0.05 (-0.06–0.03), while in the low intake group, the OR and 95% CI were−0.04 (-0.06–0.02).

**Table 4:** Interactive effect of vitamin D and dietary magnesium intake on Beta cell function is impaired.

**Variable**	**Model 1**			**Model 2**			**Model 3**		
	**OR (95%CI)**	***P*-value**	***P* for interaction**	**OR (95%CI)**	***P*-value**	***P* for interaction**	**OR (95%CI)**	***P*-value**	***P* for interaction**
VD	0.99 (0.99–0.99)	<0.001		0.99 (0.99–0.99)	<0.001		0.95(0.92–0.98)	<0.001	
**Magnesium intake group**									
<267 mg/day VD	0.99 (0.99–0.99)	<0.001	<0.001	0.99 (0.99–0.99)	<0.001	<0.001	0.96(0.93–0.99)	<0.001	<0.001
≥267 mg/day VD	0.98 (0.97–0.99)	<0.001		0.96 (0.93–0.99)	<0.001		0.94 (0.92–0.96)	<0.001	

*Model 1, Non-adjusted; Model 2, Adjusted age, gender and race; Model 3, Adjusted age, gender, race, obesity, education level, physical activity, smoking status, the season of examination, and dietary calcium intake*.

### Curve Fitting Analysis

**Figure 4:**
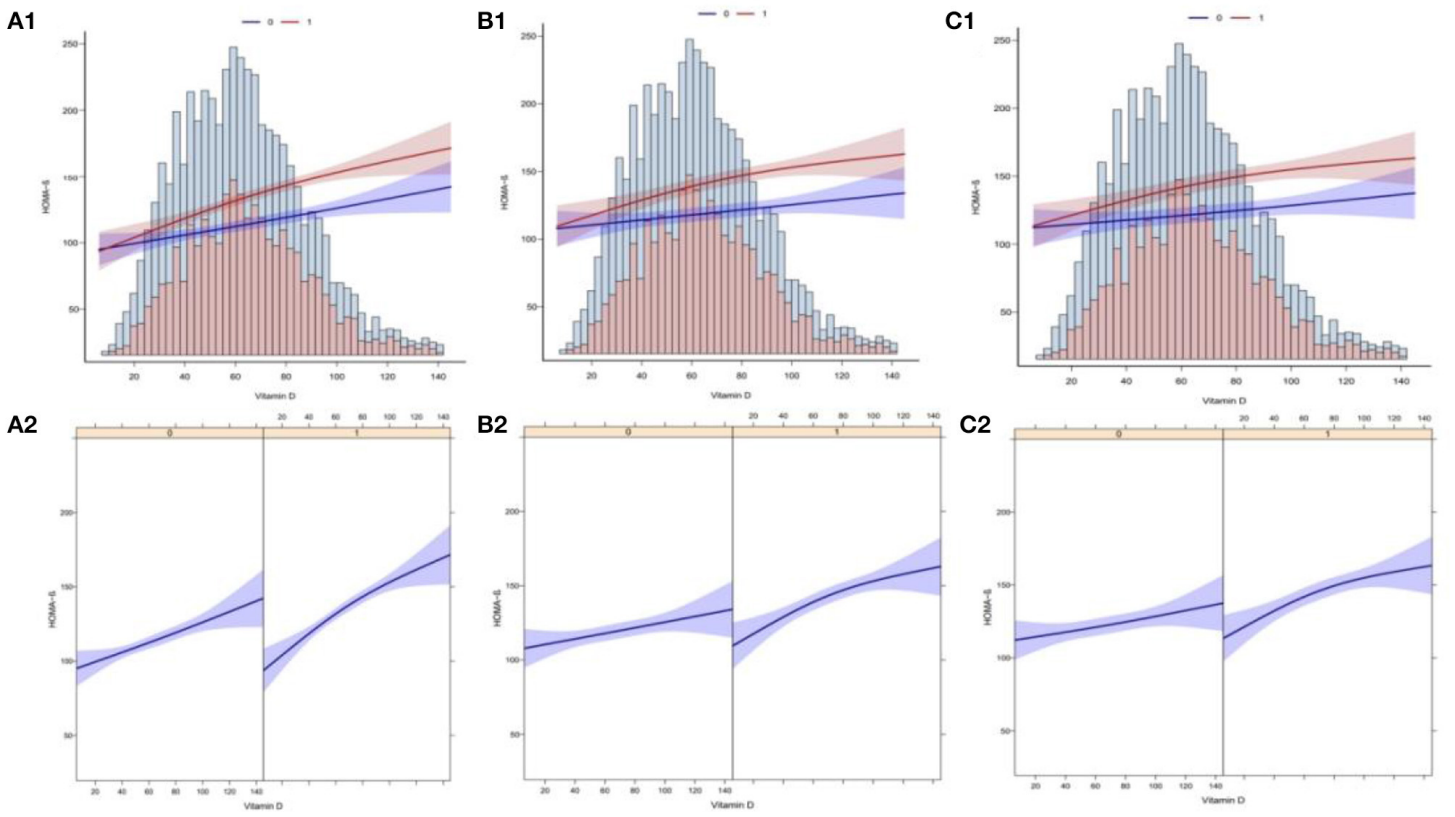
Curve fitting of Vitamin D levels and the risk of HOMA-β index. **(A1)** The association between VD and HOMA-β with no adjustment for covariates (Model 1). **(A2)** The association between VD and HOMA-β with no adjustment for covariates (Model 1). **(B1)** The association between VD and HOMA-β with the adjustment of age, gender and race (Model 2). **(B2)** The association between VD and HOMA-β with the adjustment of age, gender and race (Model 2). **(C1)** The association between VD and HOMA-β with the adjustment other covariates (Model 3). **(C2)** The association between VD and HOMA-β with the adjustment other covariates (Model 3).

In this study, we analyzed whether there was a linear correlation between Vit D and HOMA-β index at different levels of Magnesium intake. 1A and 1B display the association between Vit D and HOMA-β index based on multiple linear regression using unadjusted latent variables. 2A and 2B present the association between Vit D and HOMA-β index based on multiple linear regression after adjustment with Model 2. Meanwhile, 3A and 3B display the association between Vit D and HOMA-β index based on multiple linear regression after adjustment with complete model. To sum up, at different levels of Magnesium intake, all the correlations between Vit D level and HOMA-β index were linear (Figure 4).

## Discussion

In recent decades, the incidence of diabetes has increased tremendously (40, 41). The onset risk has presented an evident upward trend. Most countries and regions have invested substantially in the diagnosis and treatment of this disease (42). The primary factor leading to diabetes is β-cell impairment. Under the trend of growing incidence of diabetes, there are far more people with impaired pancreatic β-cell functionality (43). Therefore, how to effectively prevent β-cell impairment has also become a focus of our concern.

In this study, we built multiple linear regression models to explore the independent correlation between serum Vit D levels and HOMA-β index, and to clarify whether such correlation is affected by the dietary Magnesium intake. Apart from that, multiple logistic regression models were built to analyze the independent correlation between serum Vit D levels and pancreatic β-cell dysfunction, and to clarify whether such independent correlation is affected by the dietary Magnesium intake. After excluding potential confounding factors, it was found in the multivariate regression analysis that the serum Vit D levels was independently correlated with the HOMA-β index and pancreatic β-cell dysfunction, and that such correlations were affected by the level of Magnesium intake. The smooth fitting curves also showed that at different levels of Magnesium intake, a linear correlation was present between the serum Vit D levels and the HOMA-β index. With the increasing level of Vit D, the incidence of pancreatic β-cell dysfunction decreased.

In 2021, Lu et al. (44) conducted a prospective controlled study, finding that after adjusting laboratory indicators, anthropometric data and other factors, the active Vit D supplementation could improve the HOMA-β index in 6 months among diabetic patients. Mattla et al. (45) carried out an RCT based on the Finnish population. After adjusting for covariates such as gender, age and BMI, the pancreatic β-cells were more severely dysfunctional among those with lower Vit D levels. This may also suggest the presence of a positive correlation between pancreatic β-cell dysfunction and Vit D (45).

Research has shown that Magnesium is necessary for proper glucose utilization and insulin signaling. Pancreatic β-cells secrete insulin, and such secretion is associated with the ATP-sensitive K+ channel. However, the lack of Magnesium leads to the dysfunction of this channel, which may impair the insulin secretion (29). Takaya et al. review of previous studies found that Magnesium is vitally important for the phosphorylation of insulin receptors and the tyrosine kinase activity in insulin signaling pathways. Inhibiting the intracellular Magnesium concentration might lead to defective activity of enzymes, which also altered the insulin sensitivity by influencing the binding receptor activity or intracellular signal transduction, ultimately resulting in abnormal glucose metabolism (46). A cross-sectional study by Huang et al. among the American population in 2021 demonstrated that Magnesium is an important cofactor for the double hydroxylation of Vit D, and that the activity of Vit D binding receptors is a Magnesium-dependent process. Accordingly, high Magnesium intake can promote the activation of Vit D and increase the transfer of Vit D to target tissues (8). To sum up, Magnesium supplementation may enhance the activity of Vit D, thereby increasing its protective function on pancreatic β-cells.

However, this study still has several limitations. Firstly, due to the sample restrictions, we did not consider some special populations, such as pregnant women and children. It remains unknown whether the results of this study are applicable to these special populations. Certainly, this limitation will be resolved in the future since we will investigate these populations in future studies. Secondly, given the cross-sectional nature of this study, it is impossible to draw the causal association of Vit D level with HOMA-β and pancreatic β-cell function. To analyze this causal association, a cohort study is required in the future. Finally, our dietary data is derived from self-reported 24-h diet recalls, which inevitably has some recall and self-reporting biases. However, the impact is too small to affect our results, since NHANES suppresses these biases by collecting data with professionals and selecting subjects through a multi-stage stratified probability design. Certainly, this study also has certain advantages over others, such as the large sample size of participants. Moreover, we adopted an advanced statistical method (multiple imputation) for processing the missing data, in order to maximize the statistical power of results and minimize the errors.

## Conclusion

After adjusting for potential confounding factors, this study finds that among the US adult population, Vit D level is independently associated with the HOMA-β index and pancreatic β-cell dysfunction, and that the Magnesium intake enhances such association. Meanwhile, we also know that Vit D influences normal glucose metabolism. Besides, safe sun exposure also provides a good way for increasing the concentration of circulating Vit D and benefiting the health. As a result, among the numerous ways to enhance the concentration of circulating Vit D, increasing the intake of dietary magnesium or safe sun exposure would prove an effective way. This finding offers a new insight for the clinical research. However, we cannot determine their causality given the cross-sectional nature of this study. Hence, more RCTs or cohort studies are required in the future to confirmed the obtained finding.

## Data Availability Statement

Data can be obtained from the NHANES database (https://www.cdc.gov/nchs/nhanes/).

## Author Contributions

RG and YL conceived the idea and wrote the manuscript. GL and RG collected, read the literature, and revised the article. LY and YL read through and corrected the manuscript. All authors contributed to the article and approved the submitted version.

## Funding

This work was supported by General Project of Natural Science Foundation of Qinghai Province (2020-ZJ-930), Effects of high altitude environment and season on vitamin D, bone metabolism factors and muscle volume in adults.

## Conflict of Interest

The authors declare that the research was conducted in the absence of any commercial or financial relationships that could be construed as a potential conflict of interest.

## Publisher's Note

All claims expressed in this article are solely those of the authors and do not necessarily represent those of their affiliated organizations, or those of the publisher, the editors and the reviewers. Any product that may be evaluated in this article, or claim that may be made by its manufacturer, is not guaranteed or endorsed by the publisher.

## Abbreviations

Magnesium: Magnesium
Vit D: Vitamin D
IDF: International Diabetes Federation
T2MD: Type 2 Diabetes
US: United States
FPG: Fasting Plasma Glucose
BMI: Body Mass Index
CDC: Centers for Disease Control
MEC: Mobile Examination Center.
